# COVID-19-Ausbrüche in prekären Wohn- und Arbeitssettings: Ergebnisse eines After-Action-Reviews und Empfehlungen für den Öffentlichen Gesundheitsdienst

**DOI:** 10.1007/s00103-025-04157-8

**Published:** 2025-11-14

**Authors:** Janina Schäfer, Juliane Seidel, Maria an der Heiden, Renke Biallas, Annika Heck, Franziska Hommes, Brigitte Joggerst, Annette Jurke, Angelika Puls, Meike Schöll, Philipp Schulze, Amrei Wolter, Katja Kajikhina, Navina Sarma

**Affiliations:** 1 Fachgebiet 31: ÖGD-Kontaktstelle | Krisenmanagement, Ausbruchsuntersuchungen und Trainingsprogramme, Abteilung für Infektionsepidemiologie, Robert Koch-Institut, Seestraße 10, 13353 Berlin, Deutschland; 2 Fachgebiet 34: HIV/AIDS und andere sexuell oder durch Blut übertragbare Infektionen, Abteilung für Infektionsepidemiologie, Robert Koch-Institut, Berlin, Deutschland; 3 Fachgebiet 35: Gastrointestinale Infektionen, Zoonosen und tropische Infektionen, Abteilung für Infektionsepidemiologie, Robert Koch-Institut, Berlin, Deutschland; 4 Postgraduiertenausbildung für angewandte Epidemiologie (PAE), Abteilung für Infektionsepidemiologie, Robert Koch-Institut, Berlin, Deutschland; 5 ECDC Fellowship Programme, Field Epidemiology path (EPIET), European Centre for Disease Prevention and Control (ECDC), Stockholm, Schweden; 6 Gesundheitsamt, Landratsamt Karlsruhe, Karlsruhe, Deutschland; 7 Fachgruppe Infektionsepidemiologie, Landesamt für Gesundheit und Arbeitsschutz Nordrhein-Westfalen, Bochum, Deutschland; 8 Institut für Hygiene, Universitätsklinik Münster, Münster, Nordrhein-Westfalen Deutschland; 9 Gesundheitsamt für die Stadt und den Landkreis Göttingen, Göttingen, Deutschland; 10 Dezernat Jugend, Soziales und Gesundheit, Fachamt Gesundheit, Infektionsschutz, Bezirksamt Hamburg-Mitte, Hamburg, Deutschland

**Keywords:** Soziale Determinanten, Gesundheitliche Ungleichheit, Krisenmanagement, Aufarbeitung, Pandemieplanung, Social determinants, Health inequalities, Crisis management, Post-crisis review, Pandemic preparedness planning

## Abstract

**Hintergrund:**

Die COVID-19-Pandemie stellte den Öffentlichen Gesundheitsdienst (ÖGD) in Deutschland vor erhebliche Herausforderungen, insbesondere bei Ausbruchsgeschehen in prekären Wohn- und Arbeitssettings.

**Methoden:**

Im Rahmen eines im Mai 2023 durchgeführten After-Action-Reviews (AAR) analysierten 29 Fachkräfte aus Gesundheitsämtern und weiteren Behörden 9 Ausbrüche aus den Jahren 2020–2022, basierend auf methodischen Empfehlungen der Weltgesundheitsorganisation und des Europäischen Zentrums für die Prävention und die Kontrolle von Krankheiten. Ziel war es, Herausforderungen und erfolgreiche Ansätze in den Bereichen „Preparedness and Response“ sowie „Public Health and Social Measures“ zu identifizieren und daraus praxisnahe Handlungsempfehlungen abzuleiten.

**Ergebnisse:**

Die 2‑tägige Online-Veranstaltung ermöglichte aktive Diskussionen, die dokumentiert und anschließend mit den Teilnehmenden konsolidiert wurden. Als besonders herausfordernd wurden die behördliche Kommunikation mit Bevölkerung, Zivilgesellschaft und Unternehmen, die intersektorale Zusammenarbeit mit diversen Akteur:innen sowie die strukturelle Vorbereitung des ÖGD auf komplexe Lagen benannt. Gleichzeitig wurden zahlreiche Beispiele guter Praxis dokumentiert – etwa zur ressortübergreifenden Kooperation, zum Aufbau interner Strukturen oder zur zielgruppenspezifischen Kommunikation. Aufbauend auf diesen Erkenntnissen entwickelten die Teilnehmenden Empfehlungen zur Verbesserung des Krisenmanagements bei künftigen Ausbruchsgeschehen in prekären Settings.

**Diskussion:**

AAR stellen ein akzeptiertes und umsetzbares Instrument zur strukturierten Aufarbeitung epidemischer Lagen und zum Wissenstransfer im ÖGD dar und können somit zur besseren Vorbereitung auf zukünftige Krisen beitragen.

## Abstract (en)

**Background:**

The COVID-19 pandemic posed significant challenges for the public health service (ÖGD) in Germany, particularly during outbreaks in precarious living and working conditions.

**Methods:**

As part of an after-action review (AAR) conducted in May 2023, 29 professionals from public health offices and other authorities analyzed nine such outbreak events from 2020 to 2022 based on the methodological recommendations of the World Health Organization and the European Centre for Disease Prevention and Control. The aim was to identify key challenges and successful approaches in the areas of “preparedness and response” and “public health and social measures” and to derive practical recommendations for future action.

**Results:**

The two-day online event facilitated active discussions, which were documented and consolidated with the participants. Major challenges identified included official communication with the public, civil society, and businesses; intersectoral collaboration with diverse stakeholders; and the ÖGD’s structural preparedness for complex situations. At the same time, numerous examples of good practice were documented—such as cross-sectoral cooperation, the development of internal structures, and target group-specific communication. Based on these findings, participants developed recommendations to strengthen crisis management in future outbreaks in precarious settings.

**Discussion:**

AARs are accepted and applicable tools for structured analysis of epidemic situations and for knowledge transfer within the ÖGD and can thereby contribute to improved preparedness for future crises.

## Hintergrund

Prekäre Wohn- und Arbeitssettings sind durch Unsicherheiten hinsichtlich Dauer, Perspektive, Verlässlichkeit oder rechtlichen Status gekennzeichnet. Dazu zählen Arbeitsverhältnisse mit niedrigem Lohn, fehlender sozialer Absicherung und ungewisser Zukunft – etwa befristete Verträge, Leiharbeit, Minijobs oder Scheinselbstständigkeit [1]. Wohnen und Unterbringung gelten als prekär, wenn Menschen keinen gesicherten Wohnraum haben oder unter gesundheitlich bedenklichen Bedingungen leben. Dies kann z. B. für Beschäftigte in fleischverarbeitenden Betrieben, der Saison- und Erntearbeit, Werften und Logistikunternehmen sowie Bewohnende deprivierter Stadtteile der Fall sein. Menschen, die in solchen Settings leben und arbeiten, können von Diskriminierung, Armut, Benachteiligung, Ausgrenzung und Rassismus betroffen sein. Systematische Benachteiligung bei Bildung, auf dem Arbeitsmarkt, bei Wohnverhältnissen und der Gesundheitsversorgung steht in engem Zusammenhang mit der körperlichen und psychischen Gesundheit, arbeitsplatzbezogenen Gefährdungen, individuellem Gesundheitsverhalten und der Nutzung von Präventions- und Versorgungsangeboten [2–4]. Dies kann mit einer erhöhten Vulnerabilität dieser Menschen gegenüber Ausbrüchen von Infektionskrankheiten sowie mit einer erschwerten Umsetzung von Schutzmaßnahmen im Kontext von Ausbruchsgeschehen einhergehen.

Während der COVID-19-Pandemie traten in Deutschland SARS-CoV-2-Ausbruchsgeschehen in prekären Wohn- und Arbeitssettings auf [5, 6]. Dabei war der Öffentliche Gesundheitsdienst (ÖGD) mit spezifischen Herausforderungen in der Bewältigung dieser Ausbruchsgeschehen konfrontiert. Dazu zählten die intersektorale Zusammenarbeit, sprachliche und strukturelle Barrieren in der Kommunikation mit den Betroffenen sowie Schwierigkeiten bei der Kontaktpersonennachverfolgung aufgrund intransparenter Betriebs- und Subunternehmensstrukturen [6, 7].

Bei der retrospektiven Bewertung von Infektionsschutzmaßnahmen und dem angewandten Krisenmanagement wurden prekäre Wohn- und Arbeitssettings bislang nicht ausreichend untersucht. Um Ausbruchsgeschehen in diesen Settings und die mit ihnen verbundenen Herausforderungen strukturiert zu betrachten, wurde unter Federführung des Robert Koch-Instituts (RKI) im Mai 2023 ein After-Action-Review (AAR) mit Vertreter:innen des ÖGD durchgeführt. AAR sind qualitative und kollektive Lernprozesse zur kritischen und systematischen Analyse von Maßnahmen, die zur Bewältigung einer epidemisch bedeutsamen Lage ergriffen wurden. Sie dienen dazu, Erfahrungen aus konkreten Ereignissen aufzuarbeiten, um Stärken, Schwächen und Verbesserungspotenziale zu identifizieren und daraus praxisnahe Handlungsempfehlungen für zukünftige Krisen abzuleiten [8]. Im vorliegenden Beitrag werden die Ergebnisse aus dem AAR vorgestellt. Diese beinhalten sowohl die von den Teilnehmenden identifizierten Beispiele guter Praxis, Herausforderungen und Handlungsempfehlungen als auch Ergebnisse hinsichtlich Akzeptanz und Machbarkeit von AAR als Methode für den ÖGD in der Aufarbeitung spezifischer Lagen.

## Methoden

Der im Mai 2023 durchgeführte AAR bezog 9 SARS-CoV-2-Ausbruchsgeschehen in prekären Wohn- und Arbeitssettings ein, die sich zwischen Januar 2020 und Dezember 2022 in 6 Bundesländern in Deutschland ereigneten. Ausgeschlossen wurden Wohnsettings für geflüchtete Personen, wie etwa Gemeinschaftsunterkünfte oder Erstaufnahmeeinrichtungen, weil der Hauptfokus des AAR auf der Verschränkung von prekären Arbeits- und Wohnsettings lag und das Themenspektrum bei Einschluss von Gemeinschaftsunterkünften und Erstaufnahmeeinrichtungen für das geplante Format zu breit gewesen wäre.

Methodisch basierte der AAR auf Empfehlungen der Weltgesundheitsorganisation (WHO; [8]), des Europäischen Zentrums für die Prävention und die Kontrolle von Krankheiten (ECDC; [9, 10]) und generischen AAR-Vorlagen des RKI, wie z. B. Konzeptpapieren, Moderationsleitfäden und Dokumentationsvorlagen. Aus den durch das ECDC definierten Fokusbereichen wurden „Preparedness and Response“ und „Public Health and Social Measures“ zur Betrachtung ausgewählt. Der AAR wurde als Online-Workshop mit einer Gesamtdauer von insgesamt 7 Stunden an 2 aufeinanderfolgenden Tagen (jeweils 3,5 h) durchgeführt. Das RKI übernahm die Konzeption und Umsetzung und legte dabei Methodik und Themenschwerpunkte fest.

Im AAR wurden validitätssteigernde Überlegungen zur Verbesserung der Methoden und Berichterstattung berücksichtigt (Tab. 2 in [11]). Dazu gehörten u. a. die Orientierung an den theoretischen Rahmenwerken von ECDC und WHO, die Möglichkeit der Teilnehmenden, in Auswertungen und Publikationen einbezogen zu werden, sowie die Validierung der Ergebnisse durch Triangulation mit weiteren Datenquellen (z. B. Publikationen zu ähnlichen Ausbruchsgeschehen) sowie durch die Beteiligung mehrerer Auswertender.

Für die Auswahl der Teilnehmenden wurde zunächst eine Akteursanalyse in Anlehnung an das durch das ECDC [9] beschriebene Vorgehen durchgeführt. Ziel war es zunächst, Akteur:innen zu identifizieren, die in das Management von Ausbrüchen in prekären Wohn- und Arbeitssettings involviert waren. Aufgrund der Vielzahl der identifizierten Akteur:innen (u. a. Ministerien, Behörden, Verwaltung, Labore, Krankenhäuser, Medien, Gewerkschaften, Arbeitnehmende, Bewohner:innen, Betriebe, Sprachmittelnde, Selbstorganisationen, Schulen, Feuerwehr, Polizei, Wohlfahrtsorganisationen) wurde entschieden, nur Personen mit operativer Verantwortung und praktischer Erfahrung im Ausbruchsmanagement zu beteiligen. Im nächsten Schritt wurden die 6 Landesgesundheitsbehörden kontaktiert, in deren Zuständigkeit die 9 ausgewählten Ausbruchsgeschehen stattgefunden hatten, und um Beteiligung gebeten. Weiterhin wurden alle Gesundheitsämter zur Teilnahme eingeladen, die in mindestens einem der 9 Ausbruchsgeschehen involviert waren. Aufgrund ihrer Kenntnisse über den spezifischen Ausbruchskontext wurden sie gebeten, jeweils 2 weitere Teilnehmende (innerhalb oder außerhalb des ÖGD) für den AAR zu benennen.

### Ablauf und Durchführung

Zu Beginn des AAR wurde vereinbart, dass alle Informationen vertraulich behandelt werden und bei der Veröffentlichung der Ergebnisse keine personenbezogenen Rückschlüsse möglich sein dürfen. Der AAR begann mit einer Zeitstrahlübung, in der zentrale Meilensteine des Pandemieverlaufs und der jeweiligen Ausbruchsgeschehen im Betrachtungszeitraum visualisiert wurden. Diese Übung diente der Erinnerung und Kontextualisierung zentraler Ereignisse. Schon in diesem Stadium wurden übergreifende Muster und standortspezifische Besonderheiten sichtbar. In thematisch vorstrukturierten Kleingruppen diskutierten die Teilnehmenden Herausforderungen und gute Praxisbeispiele in den Bereichen Kommunikation, behördeninterne Prozesse, externe Kooperationen und Rahmenbedingungen. Zur inhaltlichen Schärfung wurden die Themenfelder durch das Organisationsteam angepasst und in 5 Schwerpunkte überführt: (1) Kommunikation und Zusammenarbeit mit der Bevölkerung und zivilgesellschaftlichen Akteur:innen, (2) behördeninterne Abläufe und Zusammenarbeit, (3) intersektorale Kooperation, (4) strukturelle und rechtliche Rahmenbedingungen sowie (5) settingspezifische Risiken und Umsetzung von Maßnahmen. Am zweiten Tag wurden die Ergebnisse durch die Teilnehmenden priorisiert und im Hinblick auf übertragbare Handlungsempfehlungen bearbeitet. Protokollierung sowie digitale Abstimmungstools und Whiteboards unterstützten die Diskussion und Ergebnissicherung.

Unter Nutzung von Onlinetools erfolgte zu Beginn des Workshops eine anonyme Abfrage der Erwartungen für den AAR und am Tagesende jeweils eine Feedbackrunde mit den Teilnehmenden sowie ein „Hot Debriefing“ innerhalb des Organisationsteams. So konnten Anpassungen für den zweiten Workshop-Tag vorgenommen werden. Ein abschließendes internes Treffen des Organisationsteams diente der methodischen Reflexion. Nach Auswertung der Ergebnisse wurde ein interner Abschlussbericht erstellt und durch die Teilnehmenden kommentiert und ergänzt.

## Ergebnisse

Alle 6 Landesbehörden stimmten einer Teilnahme ihrer Gesundheitsämter am AAR zu. Insgesamt nahmen 29 Personen unterschiedlicher Erfahrungsstufen und beruflicher Hintergründe teil: aus allen 11 angefragten Gesundheitsämtern, aus 3 Landesbehörden, einer kommunalen Ordnungsbehörde und dem RKI.

### Beispiele guter Praxis

Im Rahmen des AAR identifizierten die Teilnehmenden eine Vielzahl von Beispielen guter Praxis, die sich als förderlich für das Management von Ausbruchsgeschehen in prekären Wohn- und Arbeitssettings erwiesen hatten.

Im Themenfeld Kommunikation und Zusammenarbeit der Behörden mit der Bevölkerung und der Zivilgesellschaft wurden insbesondere der kontinuierliche Austausch mit etablierten Kontakten und deren Pflege hervorgehoben. Konkret benannt wurde hier der Kontakt zu zivilgesellschaftlichen Akteur:innen (Interessenvertretungen, Nichtregierungsorganisationen, Sozialarbeitende, Sprachmittler:innen). Dieser erwies sich als zentral für eine effektive Kommunikation von Infektionsschutzmaßnahmen und stärkte die Zusammenarbeit mit diesen Akteur:innen. Des Weiteren als hilfreich benannt wurden mehrsprachige Mitarbeitende innerhalb der Behörden selbst, die Vorhaltung aktueller Sprachmittler:innenlisten, Übersetzungen von Verordnungen und Erstellung zielgruppenspezifischer Informationsmaterialien (z. B. Aushänge, Informationsvideos). Als wirksam wurde zudem die frühzeitige Etablierung klarer Kommunikationswege beschrieben, z. B. durch Dokumentation mündlicher Absprachen oder den Einsatz eines Kommunikationsteams aus Sprachmittler:innen, Sozialarbeitenden und ÖGD-Fachkräften.

Hinsichtlich der behördeninternen Zusammenarbeit wurden eine hohe intrinsische Motivation der Mitarbeitenden, Entgegenwirken der Überlastung der Mitarbeitenden durch transparente und wertschätzende Führungsstrukturen sowie klar definierte Rollen und Aufgaben und nachhaltige Dokumentation als zentrale Erfolgsfaktoren benannt. Die Umverteilung personeller Ressourcen und die Möglichkeit zur Delegation von Routineaufgaben innerhalb der Behörden trugen maßgeblich zur Bewältigung der hohen Arbeitsbelastung bei. Die pandemiebedingte Repriorisierung von Aufgaben erlaubte es, Ressourcen gezielt auf das Ausbruchsgeschehen zu fokussieren. Darüber hinaus erwiesen sich regelmäßige Lagebesprechungen ebenso wie die frühzeitige Einbindung der Presse- und Öffentlichkeitsarbeit als förderlich. Die regelmäßige Durchführung von Routine- und *Ad-hoc*-Schulungen für Mitarbeitende im Infektionsschutz wurde ebenfalls als Beispiel guter Praxis benannt.

Im Bereich der intersektoralen Zusammenarbeit wurden regelmäßig tagende Krisenstäbe sowie ein strukturierter Austausch zwischen Akteur:innen unterschiedlicher Sektoren (u. a. Arbeitsschutz, Sozialbehörden und -beratung, Ordnungsbehörden) und Verwaltungsebenen (lokal, Landes- und Bundesebene) als effektive Strategien identifiziert. Auch behördenübergreifende Unterstützungsangebote wie der Einsatz von Containment Scouts wurden als hilfreiche Strategie hervorgehoben. Darüber hinaus identifizierten die Teilnehmenden Pandemie- und Krisenpläne auf kommunaler, Länder- oder nationaler Ebene als wichtige Erfolgsfaktoren in der Bewältigung der Ausbruchsgeschehen. Die Nutzung von Erfahrungen aus Pandemieübungen trug aus Sicht der Teilnehmenden maßgeblich zu einer erfolgreichen Krisenreaktion bei.

Bezüglich der Rahmenbedingungen wurde die pandemiebedingt erhöhte Bereitstellung personeller, finanzieller und materieller Ressourcen als maßgeblich für die effektive Bewältigung der Ausbruchsgeschehen benannt.

### Herausforderungen

Die Teilnehmenden beschrieben eine Vielzahl struktureller und operativer Herausforderungen, mit denen der ÖGD bei der Bewältigung von Ausbrüchen in prekären Wohn- und Arbeitssettings konfrontiert war. Ursächlich hierfür waren unter anderem die Neuartigkeit und das Ausmaß der Ereignisse während der COVID-19-Pandemie, die mit einem erheblichen öffentlichen Interesse sowie einem Erwartungsdruck vonseiten politischer Entscheidungstragender einhergingen. Die hohe Situationsdynamik führte zu mehrfachen Änderungen von Erlasslagen und Verordnungen, die teils kurzfristig an die Gesundheitsämter und Landesbehörden kommuniziert wurden. Die Teilnehmenden berichteten, dass dadurch oft nur begrenzt Zeit für die organisatorische Vorbereitung und Umsetzung blieb und teilweise Unsicherheit bezüglich juristischer Rahmenbedingungen zur Umsetzung bestand.

Besondere Schwierigkeiten ergaben sich durch die häufig unübersichtliche Unterbringungssituation von Personen in prekären Wohn- und Arbeitsverhältnissen – etwa durch überbelegte Unterkünfte für Beschäftigte, fehlende Transparenz und Dokumentation der Anstellungsverhältnisse sowie überfüllte und prekäre Wohngebäude. Dies erschwerte die Kontaktpersonennachverfolgung und die Durchsetzung und Kontrolle von Infektionsschutzmaßnahmen. Die Teilnehmenden berichteten zudem, dass die Kommunikation und Zusammenarbeit mit den betroffenen Personen möglicherweise durch ein niedriges Vertrauen der Betroffenen in staatliche Stellen sowie durch die wirtschaftliche Abhängigkeit von instabilen Beschäftigungsverhältnissen erschwert waren. Maßnahmen wie Quarantäne oder Isolation können für die betroffenen Personen ein gravierendes ökonomisches Risiko darstellen, was möglicherweise zu einer eingeschränkten Adhärenz gegenüber behördlichen Anordnungen beitrug.

Ein weiterer von den Teilnehmenden als herausfordernd benannter Aspekt war der Gebrauch von sozialen Netzwerken oder Chat-Diensten. Diese Kommunikationskanäle standen im Konflikt mit datenschutzrechtlichen Anforderungen innerhalb der Behörden und es gab wenig Erfahrung mit ihrer Nutzung. Hinzu kam, dass niedrigschwellige Unterstützungsstrukturen, die vor der Pandemie als wichtige Multiplikatoren für Gesundheitsinformationen fungierten (z. B. aufsuchende Sozialarbeit), pandemiebedingt nur eingeschränkt oder gar nicht operierten. Auch fehlten in einigen Fällen Ressourcen für eine adäquate Sprachmittlung, was die Informationsweitergabe und den Abbau von Ängsten und möglichen Vorbehalten erschwerte.

Ein weiterer kritischer Aspekt betraf die Nutzung und Verfügbarkeit von Quarantäneeinrichtungen. Diese wurden teilweise erst verspätet eingerichtet oder früh wieder geschlossen, wodurch ihre Wirksamkeit in der Unterbrechung von Infektionsketten eingeschränkt war. Darüber hinaus wurde die Einhaltung von Quarantänemaßnahmen, bedingt durch die schwierigen Unterbringungs- und Wohnverhältnisse und prekären Beschäftigungsverträge, von den Teilnehmenden als teilweise unzureichend und schwer überprüfbar eingeschätzt. Gleichzeitig wurde ein mangelndes Bewusstsein – sowohl innerhalb von Behörden als auch bei externen Akteur:innen – für die psychische Belastung langer Quarantänezeiten als Herausforderung benannt. Eine geringe Bereitschaft seitens einzelner Akteur:innen, psychosoziale Aspekte in die Maßnahmenplanung zu integrieren, wurde als hinderlich bewertet.

Die intersektorale Zusammenarbeit wurde von den Teilnehmenden insbesondere dann als problematisch erlebt, wenn sich aus konkurrierenden oder unklaren Zuständigkeiten Zielkonflikte ergaben – etwa zwischen Arbeitsschutz, Infektionsschutz und Wirtschaftsunternehmen. Die Interessen einzelner Wirtschaftsakteur:innen standen dabei mitunter im Widerspruch zu epidemiologischen Erfordernissen. Die Kooperationsbereitschaft mancher Unternehmen in Bezug auf den Infektionsschutz wurde als gering beschrieben. Teilweise kam es zum Versuch, Entscheidungen des Gesundheitsamtes zu umgehen, wodurch die Umsetzung notwendiger Infektionsschutzmaßnahmen erschwert wurde. Teilnehmende berichteten, dass die Kommunikation mit Unternehmen und Subunternehmen eine Herausforderung darstellte, da Ansprechpersonen und Zuständigkeiten nur schwer zu identifizieren oder zu erreichen waren.

Innerhalb der Verwaltung wurden in einigen Fällen mangelnde Abstimmung zwischen verschiedenen Ebenen und Behörden sowie unzureichende Klärung von Zuständigkeiten benannt. Die Teilnehmenden betonten, dass es an niedrigschwelligen, institutionenübergreifenden *Ad-hoc*-Austauschformaten fehlte, die den Transfer bewährter Vorgehensweisen und den regelmäßigen Informationsaustausch zwischen den Gesundheitsämtern und mit anderen Behörden hätten fördern können.

Auch innerhalb der Behörden wurden Herausforderungen deutlich. Die Teilnehmenden stellten fest, dass die personellen Ressourcen der Gesundheitsämter teilweise nicht ausreichend waren, um in der Krisenlage adäquat reagieren zu können. Die Rekrutierung von geeignetem Personal stellte eine der größten Herausforderungen dar, wobei die Beantragung zusätzlicher personeller Ressourcen nicht immer rechtzeitig oder vollständig umgesetzt wurde. Dennoch reichte das vorhandene Personal nicht aus, weshalb in einigen Fällen eine Unterstützung durch Ressorteinrichtungen wie der Bundeswehr oder des RKI erfolgte.

Ebenfalls als herausfordernd beschrieben wurde die strukturelle und inhaltliche Vorbereitung des ÖGD auf Ausbruchsgeschehen in prekären Settings. Einige Teilnehmende verwiesen auf das Fehlen spezifischer Schulungen und Weiterbildungen sowie Krisenpläne (bzw. deren eingeschränkter Anwendbarkeit auf Infektionsgeschehen in prekären Settings), standardisierter Handlungsanweisungen sowie differenzierter Zuständigkeitsregelungen in diesem Bereich.

### Handlungsempfehlungen

Die durch die Teilnehmenden erarbeiteten Handlungsempfehlungen lassen sich in 3 wesentliche Handlungsfelder gliedern: Kommunikation und Netzwerkarbeit, Koordination und Zusammenarbeit sowie strukturelle und organisatorische Voraussetzungen.

In Bezug auf die Kommunikation mit der betroffenen Bevölkerung und zivilgesellschaftlichen Gruppen wurde hervorgehoben, dass Vertrauen nur durch kontinuierliche Einbindung relevanter Akteur:innen aufgebaut werden kann (wie z. B. Vorarbeiter:innen, Lehrkräfte, religiöse Schlüsselpersonen). Wo möglich und sinnvoll, sollten diese Netzwerke auch außerhalb von Krisen etabliert und gepflegt werden, um eine die betroffenen Communitys einbeziehende Arbeitsweise im ÖGD zu ermöglichen. Eine transparente, mehrsprachige Informationsvermittlung wurde als essenzielle Voraussetzung für den Infektionsschutz in prekären Wohn- und Arbeitssettings identifiziert. Die Nutzung vielfältiger Kommunikationswege, einschließlich visueller und audiovisueller Formate, und zielgruppengerechte Krisenkommunikation sollten verstärkt implementiert werden. Der datenschutzkonforme Einsatz technischer Hilfsmittel (z. B. Dolmetschdienste) erfordert regelmäßige Schulungen der ÖGD-Mitarbeitenden und entsprechende Ressourcen. Auch die institutionelle Verankerung von Diversität wurde als wichtiges Handlungsfeld genannt.

Ein weiteres zentrales Thema ist die Verbesserung der Kommunikation und Koordination zwischen den verschiedenen Ebenen und Sektoren. Hier wurde eine stärkere Rückkopplung zwischen lokaler Verwaltung und politischen Entscheidungstragenden empfohlen, um die Praktikabilität von Verordnungen zu sichern. Die Teilnehmenden empfahlen, einen regelmäßigen operativen und strategischen Austausch zwischen öffentlichen Institutionen, sozialen Trägern sowie zwischen Gesundheitsämtern zu etablieren. Der Transfer von Wissen und Erfahrungen aus vorherigen Krisen sollte institutionell verankert werden, z. B. durch niedrigschwellige digitale Austauschplattformen und standardisierte und nachhaltige Dokumentationssysteme.

Die Teilnehmenden hoben die Notwendigkeit struktureller Verbesserungen hervor. Dies umfasst den Aufbau von Netzwerken mit klar definierten Zuständigkeiten, die Einbindung sozialmedizinischer Unterstützungsangebote in den Infektionsschutz sowie die systematische Berücksichtigung sozialer Kontexte mit besonderen Belastungsfaktoren in Pandemie- und Krisenplänen. Die Teilnehmenden sprachen sich für die Etablierung flexibler Strukturen aus, wie etwa situativer Krisenstäbe und Konzepte zur raschen Mobilisierung personeller Ressourcen. Darüber hinaus betonten sie die Bedeutung von einsatzbereiten Formaten und Konzepten, die im Falle eines Ausbruchsgeschehens aktiviert werden können. Dazu zählen z. B. Checklisten, Arbeitsanweisungen und regelmäßig aktualisierte Kontaktlisten mit Zuständigkeiten und Funktionen innerhalb und außerhalb der Behörden. Die Teilnehmenden empfahlen Intra-Action-Reviews (IAR) – also Lernprozesse während laufender Krisen – als Chance, um frühzeitig strategische und operative Anpassungspotenziale zu erkennen.

In 2 Feedbackrunden hatten die Teilnehmenden die Möglichkeit, Rückmeldungen zum AAR zu geben. Insbesondere wurde die Möglichkeit des überregionalen Erfahrungsaustauschs und der Netzwerkbildung durch den AAR selbst positiv hervorgehoben. Zudem wurde der Wunsch nach konkreten Tools und Checklisten bei großen Ausbrüchen geäußert. Manche Teilnehmende empfanden das Format als zu kurz und hätten gerne mehr Zeit für die Gruppenarbeiten gehabt. Die zu Beginn des Workshops erfragten Erwartungen der Teilnehmenden wurden erfüllt. Dies konnte auch im Debriefing des Organisationsteams bestätigt werden.

## Diskussion

AAR ermöglichen eine methodisch gestützte Auswertung von Krisenerfahrungen im ÖGD und lassen sich flexibel an vorhandene Strukturen und Ressourcen anpassen. Die hohe Bereitschaft für eine Teilnahme am AAR, die engagierten Diskussionen sowie die Ergebnisse aus den Kleingruppen und der Prozessbewertung zeigen, dass der AAR ein geeignetes Format war, um die ausgewählten SARS-CoV-2-Ausbruchsgeschehen im Hinblick auf spezifische Herausforderungen sowie Handlungsbedarfe im Umgang mit prekären Settings auf operativer Ebene strukturiert zu reflektieren.

Aufgrund der begrenzten zeitlichen Ressourcen der Mitarbeitenden im ÖGD, und um eine bundesweite Beteiligung zu ermöglichen, hatten wir uns für ein möglichst kurzes Online-Format an 2 halben Tagen entschieden. Entgegen unserer Erwartung wurde uns zurückgemeldet, dass es mehr Zeit gebraucht hätte, um noch tiefer in die Reflexion zu gehen. Zudem wäre es wünschenswert gewesen, eine breitere Vielfalt von Perspektiven einzubinden. Die fehlende Beteiligung von Akteur:innen außerhalb der Behörden stellte eine wichtige Limitation für den durchgeführten AAR dar. Sie war dadurch begründet, dass viele unterstützende Strukturen, wie z. B. Sprachmittlungsdienste und interkulturelle Teams, nach der Pandemie nicht weitergeführt wurden.

Die Teilnehmenden nannten zahlreiche Faktoren als Grundlage wirksamen Handelns in dynamischen Lagen. Besonders hervorgehoben wurden die Themen bedarfsgerechte Kommunikation und intersektorale Zusammenarbeit. Ein weiterer zentraler Aspekt waren die klare Definition von Zuständigkeiten und die Etablierung von Strukturen zur systematischen Informations- und Wissensweitergabe. Die Teilnehmenden betonten, dass prekäre Lebenssituationen in der Krisenvorbereitung stärker berücksichtigt werden müssen – etwa in Bezug auf Krisenpläne auf kommunaler, Bundesland- und nationaler Ebene sowie bei Schulungen und Weiterbildungen zum Infektionsschutz. Als Voraussetzungen für ein erfolgreiches Management von Ausbrüchen in prekären Settings wurden auch strukturelle Bedarfe des ÖGD benannt, u. a. die Verankerung dieser Aufgaben in den ÖGD-Gesetzen der Länder, die Rekrutierung geeigneten ÖGD-Fachpersonals und die Schaffung von Möglichkeiten zur datenschutzkonformen Nutzung digitaler, zielgruppenadaptierter Tools. Niedrigschwellige Austauschplattformen und kollegiale Netzwerke wurden als potenzielle Bausteine für nachhaltiges (über-)organisationales Lernen im ÖGD hervorgehoben. Um die von den Teilnehmenden identifizierten Handlungsempfehlungen umsetzen und entsprechende Kapazitäten stärken zu können, bedarf es der Ressourcenallokation und der Unterstützung durch die Leitungsfunktionen auf lokaler und nationaler Ebene, z. B. auch durch die Verankerung dieser Aufgaben in den ÖGD-Gesetzen der Länder.

Die im AAR benannten Herausforderungen stehen in Zusammenhang mit Armut sowie struktureller und institutioneller Diskriminierung in den Bereichen Arbeiten und Wohnen. Ihre Bewältigung erfordert politische Maßnahmen zur Verbesserung der Lebens- und Arbeitsbedingungen der betroffenen Menschen und die entsprechende Adaption von Gesetzen und Verordnungen – z. B. zur Verantwortlichkeit von (Sub‑)Unternehmen in Bezug auf den Infektionsschutz. Eine evidenzbasierte Ausrichtung ist dabei zentral und sollte soziale Determinanten von Gesundheit sowie ungleich verteilte Auswirkungen von Infektionsschutzmaßnahmen auf Menschen in prekären Wohn- und Arbeitsverhältnissen systematisch berücksichtigen. In den vergangenen Jahren wurde umfassend zu den Auswirkungen sozialer Ungleichheit im Kontext der COVID-19-Pandemie geforscht, wobei auch prekäre Wohn- und Arbeitssettings und deren Schnittstellen mit den Themenfeldern Migration, Armut und Diskriminierung in den Blick genommen und entsprechende Empfehlungen formuliert worden sind [3, 4, 6, 12–17]. Der durchgeführte AAR trägt zu dieser Evidenzbasis bei, indem er die in der Literatur beschriebenen Problemstellungen und Lösungsansätze durch die Perspektiven und konkreten Erfahrungen aus dem deutschen ÖGD ergänzt. Diese Empfehlungen müssen in der interpandemischen Phase in konkrete, langfristige Maßnahmen überführt werden, um gesundheitliche Ungleichheiten wirksam zu verringern und Menschen in prekären Wohn- und Arbeitsbedingungen vor und in Ausbruchsgeschehen besser zu schützen.

Die Durchführung von AAR als Evaluationsinstrumente hat in den vergangenen Jahren in Public-Health-Institutionen weltweit an Bedeutung gewonnen. Da diese Formate häufig organisationsintern durchgeführt werden und teilweise sensible Informationen enthalten, werden sie oftmals nicht publiziert. Dadurch kommt es zu einer eingeschränkten Verfügbarkeit der Ergebnisse für andere Akteur:innen. AAR sind kein standardisiertes Erhebungsinstrument, sondern zielen primär darauf ab, die spezifischen Erfahrungen und Perspektiven der Teilnehmenden abzubilden, welche sehr heterogen sein können. Dadurch ist nicht zwingend eine Generalisierbarkeit der Ergebnisse für andere Kontexte gegeben. AAR stellen dennoch ein komplementäres, lernorientiertes Format dar, das praxisnahe Einblicke in konkrete Herausforderungen und Vorgehensweisen bietet und so zur Weiterentwicklung von Strukturen und Prozessen im Krisenmanagement beitragen kann.

Teilnehmende und Organisator:innen bewerteten den durchgeführten AAR als wertvoll und gewinnbringend. Im AAR wurden Erfolg versprechende Ansätze und Herausforderungen im Krisenmanagement identifiziert. Die daraus abgeleiteten Handlungsempfehlungen bieten ergänzend zu Erkenntnissen aus Forschung und Literatur eine Grundlage für die Verbesserung des Ausbruchsmanagements in prekären Wohn- und Arbeitssettings. Die vorliegenden Ergebnisse legen nahe, dass AAR ein hilfreiches Instrument zur strukturierten Reflexion und zum organisationsübergreifenden Lernen im ÖGD darstellen. Durch diese Formate lassen sich praxisnahe und handlungsorientierte Erkenntnisse für zukünftige Lagen gewinnen und Verbesserungsbedarfe identifizieren. AAR eignen sich dabei nicht nur für die Aufarbeitung von Großereignissen, sondern auch für kleinere Lagen oder Ausbruchsgeschehen auf lokaler Ebene. Vor diesem Hintergrund ist es empfehlenswert, AAR als Bestandteil der Qualitätssicherung und Krisennachsorge im ÖGD weiter zu erproben. Sie stellen eine strukturierte, partizipative und lernorientierte Methode dar, um Wissen zu sichern, Arbeitsabläufe zu verbessern und die Resilienz des ÖGD zu stärken.

## Data Availability

Die während des After-Action-Reviews erzeugten und analysierten Daten werden in dieser Publikation aus Gründen der Vertraulichkeit des Formates und des Datenschutzes nur in aggregierter Form veröffentlicht, sodass u. a. keine Rückschlüsse auf einzelne Personen oder Institutionen möglich sind.
